# Experimental Study on Mechanical Performance and Blast Resistance of Aramid, Carbon, and UHMWPE Fabrics

**DOI:** 10.3390/polym18050612

**Published:** 2026-02-28

**Authors:** Jiang Xie, Jinzheng Liu, Hanyuan Pan, Chao Jiang, Binyuan Gao, Yilun Jiang, Zhenyu Feng

**Affiliations:** 1 Science and Technology Innovation Research Institute, Civil Aviation University of China, Tianjin 300300, China; 2 Key Laboratory of Civil Aviation Airworthiness Technology, Civil Aviation Administration of China, Tianjin 300300, China; 2023092145@cauc.edu.cn (J.L.);; 3 College of Safety Science and Engineering, Civil Aviation University of China, Tianjin 300300, China; 4 China Aero-Polytechnology Establishment, Aviation Industry Corporation of China, Beijing 100028, China; 5 Xi’an Aisheng Technology Group Co., Ltd., Xi’an 710065, China

**Keywords:** high-performance fiber fabrics, strain-rate effect, dynamic response, failure modes, overpressure attenuation

## Abstract

This study investigates the mechanical performance and blast resistance of high-performance aramid, carbon, and ultra-high molecular weight polyethylene (UHMWPE) fiber fabrics, responding to the need for lightweight and flexible materials in anti-explosion containers for aviation and critical infrastructure. The experimental methodology integrated quasi-static and dynamic tensile tests to characterize the strain-rate effect, followed by near-field air blast tests on both single-material and hybrid multi-ply fabric specimens to analyze their dynamic response, failure modes, and overpressure attenuation. Key findings revealed that carbon fabric exhibited high stiffness but was strain-rate insensitive and susceptible to brittle perforation failure, whereas aramid and UHMWPE fabrics demonstrated strain-rate sensitivity, with UHMWPE showing superior ductility and energy absorption. The hybrid multi-ply configuration (A-C-U sequence) achieved the least amount of failure, effectively utilizing the wave impedance of aramid fabric for initial shock reflection, high stiffness of carbon fabric for stress homogenization, and plasticity of UHMWPE fabric for energy dissipation. Additionally, all fabrics attenuated peak overpressure by over 80%, with enhancement observed for increased thickness. The study concludes that the strategic layering of different fabrics creates a synergistic effect, mitigating the weaknesses of individual fabrics and establishing an effective design paradigm for advanced blast-resistant structures, further enhancing the protective performance.

## 1. Introduction

With the improvement of global public security standards, modern high-density public places such as airplanes, urban rail transit stations, and large passenger hubs have put forward stricter requirements for protective equipment. Developing anti-explosion containers that can withstand extreme impact loads, such as improvised explosive devices (IEDs), could effectively improve the security of public places. Conventional anti-explosion containers predominantly employ metallic materials, which exhibit inherent limitations including excessive weight and high density, thereby unsatisfying modern lightweight requirements. Meanwhile, metallic containers subjected to implosion loading could generate hazardous fragmentation, causing secondary threats to personnel and critical infrastructure in proximity. These limitations highlight the necessity for lightweight and flexible materials [1,2,3].

With the advancement of materials science, high-performance fiber fabrics with low density and high specific strength, especially aramid and ultra-high molecular weight polyethylene (UHMWPE) fabrics, have attracted widespread attention for outstanding performance in the field of impact protection [4]. At present, the failure modes and energy absorption mechanisms of fabrics under ballistic impact have been revealed. When a projectile impacts a piece of fabric, energy is absorbed through various mechanisms, including tension in the yarns, fabric deformation, energy dispersion through frictional sliding (yarn/yarn and projectile/yarn), yarn breakage, and yarns pulling out [5,6,7,8,9]. Therefore, a comprehensive understanding of fabrics’ response under ballistic impact has been established through extensive research.

However, current research on the blast resistance of fabrics is still relatively limited, and there are few papers and reports available for reference. From 2008 to 2015, the European Union developed a lightweight explosion-proof container known as fly bags, specifically designed for the cargo hold and cabin of aircraft. The innovative solution combined fabric materials such as aramid and polyethylene. The results indicated that fly bags could effectively minimize the damage caused by implosion loading to aircraft structures [10,11]. Zangani et al. [12] proposed a method of using a combination of new textile materials and composite materials to achieve high flexibility, strength, and a flexible luggage container design, which was lightweight and could be used in both wide and narrow body aircraft. Masi et al. [13] conducted numerical analysis to investigate the influence of Kevlar fabric as the inner lining of the cabin on the structural response of the fuselage under implosion loading. The results indicated that Kevlar fiber improved the blast resistance performance of the fuselages. Xie et al. [14] investigated the failure mechanism of the polyurea-coated aramid fabric (PCAF) subjected to an air-blast loading experimentally. The results showed that perforation failure was the main failure mode of aramid fabrics. And the failure modes of PCAF mainly included fracture and exfoliation, with both weft and warp yarns breakage and polyurea failure. Based on the existing studies, a systematic understanding and investigation into the failure modes and energy absorption mechanisms of fabrics under explosion is critically needed.

Under impact loads, tensile-dominant failure serves as the principal mode of energy dissipation. Therefore, researchers have conducted a series of mechanical tests in order to understand the properties of high-performance fiber fabrics under different strain rates. Shim et al. [15] demonstrated the strain-rate effect of Twaron^®^ fabric, showing increased peak stress and elastic modulus but reduced failure strain at higher strain rates. Seidt et al. [16] found Young’s modulus of Kevlar 49 remained strain rate insensitive, while Zhu et al. [17,18] reported obvious strain-rate insensitivity for Kevlar 29, with Young’s modulus, strength, and toughness all increasing with strain rates. Yao et al. [19] quantified the rate effects across multiple fabrics, including glass, basalt, carbon, and aramid fabrics, revealing 24–59% strength increases. Several studies have specifically examined impregnated fabrics. Liu et al. [20] observed that STF-treated Kevlar fabric maintained the four-stage tensile behaviors of neat fabric in quasi-static tensile tests but showed strain-rate sensitivity in dynamic tensile tests. Liu et al. [21] found STF impregnation reduced the degree of tensile fracture in 2D/3D Kevlar fabrics. However, in current research, the strain rates applied to fabrics remain limited to relatively low values. There has been less systematic investigation of fabrics’ dynamic response under the conditions of higher strain rates.

Based on the properties of different fabrics, recent studies have systematically investigated the influence of fabric-layering position on the impact resistance of the protective structure. It aimed to optimize the impact resistance of protective structures through the strategic positioning of fabrics, leveraging their respective mechanical properties to achieve a reasonable and effective energy absorption mechanism. Patel et al. [22,23] conducted numerical studies on blast-resistant structures, focusing on two material systems. In the first study, honeycomb sandwich panels exhibited superior blast mitigation compared to equal-mass solid and stiffened panels, achieving 32–87% higher energy absorption and 34–58% lower deflection, with performance further enhanced by hybridization with carbon fiber composites. In the second study, AA7075-T6 aluminum alloy showed the highest deformation resistance among equal-mass alloys. When reinforced with a carbon/epoxy composite to form a carbon fiber-reinforced aluminum laminate (CRALL), the hybrid structure reduced peak deflection by 16.3% and increased energy absorption by 29.3% compared to the monolithic alloy plate. These works demonstrate the effectiveness of composite hybridization in optimizing lightweight protective structures under blast loading. Lu et al. [24] experimentally proved that UHMWPE/carbon interlayer hybrids achieve superior heat resistance while maintaining high strength compared to other configurations. Zhao et al. [25] quantified 42–44% improvements on tensile stiffness and strength for carbon/UHMWPE hybrids versus pure carbon fabrics, demonstrating that the hybrid design could improve the impact performance of protective structures. Li et al. [26,27] systematically compared five hybrid laminates through impact testing and FEA, identifying UHMWPE/Kevlar configurations with Kevlar layers as the impact side having the highest energy absorption (primarily through delamination and fiber breakage), followed by carbon/Kevlar. Ralph et al. [28] proposed velocity-dependent performance, showing multi-layer para-aramid/UHMWPE systems exhibit increasing specific energy absorption (SEA) with layer count at low velocities but remarkable SEA reduction at high velocities. Zhang et al. [29] investigated carbon fiber-reinforced stainless-steel tubes (CFR-SST) under combined blast and fragment loading. The 90° winding structure reduced the circumferential expansion rate by 39.2% compared to pure metal tubes. An optimized multi-angle hybrid winding strategy was further developed, achieving a 15.3% lower expansion rate than the uniform 90° structure. Hosseini et al. [30] examined CFRP and AFRP for strengthening RC columns under blast loading. Numerical validation showed CFRP reduced residual displacement, while AFRP offered superior blast resistance and energy absorption, particularly in controlling mid-span displacement. Collectively, these studies confirmed that a hybrid structure enables property combinations unattainable by single materials and that position layering critically governs energy dissipation mechanisms. Despite extensive studies on ballistic impacts, few studies have focused on the influence of the placement of different types of fabrics under explosion on the structural blast resistance.

Although fabric materials have excellent impact resistance and the ability to reduce damage, recent studies revealed shock wave–fabric interactions that may paradoxically amplify pressures. Zhu et al. [31] documented the impact enhancement phenomenon that fabrics increased rather than reduced body armor-to-body pressure transmission. It was corroborated by human trials showing higher thoracic cavity pressures in vest-wearing subjects versus unprotected ones [32]. Thom et al. [33] identified two key amplification factors, surface density and permeability. Nian et al. [34,35] conducted experimental and numerical analyses on UHMWPE fabric anti-explosion walls, revealing that transmitted pressure peaks correlate with the dynamic deformation rate, demonstrating strain-rate dependent wave transmission behavior. Naiman et al. [36] demonstrated that increased textile permeability enhances shock wave dissipation through pore channels, though their study revealed an optimal porosity threshold for peak pressure transmission, while no universal minimum-pressure porosity exists. In summary, these findings established that fabrics could unexpectedly amplify pressures under certain conditions, and the permeability and deformation dynamics of fabric govern wave transmission. When anti-explosion containers are made of fabric, the attenuation effects between fabrics and the surrounding environment must be carefully evaluated, as residual high pressures on the fabric’s back side may still pose hazards to personnel and equipment. Therefore, it necessitated a systematic investigation of the effect of overpressure attenuation.

Current research has extensively investigated the ballistic and blast resistance of fiber-reinforced laminates. While more attention has been directed toward the dynamic response behavior of fabrics under ballistic impact, systematic investigation of blast resistance mechanisms remained insufficiently revealed. In order to make up for the existing research gaps, the study systematically implemented experimental methodology to investigate the blast resistance of fabrics. Firstly, quasi-static and dynamic tensile tests were conducted to evaluate the strain-rate effect of aramid, carbon, and UHMWPE fabrics. Subsequently, a near-field air blast test was performed to research the dynamic response and failure modes of single-material and specially designed hybrid multi-ply fabrics. Finally, the effect of fabrics on overpressure attenuation was quantitatively evaluated through peak overpressure, arrival time, and positive pressure duration. In summary, this study provides a comprehensive understanding of the blast resistance performance through establishing the relationship between mechanical properties and failure modes of fabrics.

## 2. Experimental Methodology

### 2.1. Preparation of Fabric Materials

In this study, three commercially available high-performance fiber fabrics were investigated: aramid (F-268), carbon (HF30S-12K), and UHMWPE (ZTZ-24) fabrics. The fundamental parameters of yarn and fabric, including yarn linear density, bulk density, and other mechanical properties, were obtained from manufacturer specifications, as shown in Table 1. Material characterization strictly adhered to the standards, for aramid and UHMWPE fabrics, testing yarn linear density was determined according to GB/T 14343-2008, while testing bulk density followed GB/T 1033.1-2008. Mechanical properties, including yarn elongation at break and tensile modulus, were tested per GB/T 19975-2005. Carbon fabric characterization employed GB/T 3364-2008 for testing yarn linear density, GB/T 30019-2013 for yarn bulk density measurement, and GB/T 3362-2017 for testing yarn elongation at break and yarn tensile modulus. These testing standards ensured consistent and comparable mechanical properties across specimens [37,38,39,40,41,42].

### 2.2. Quasi-Static Tensile Test

#### 2.2.1. Preparation of Quasi-Static Tensile Testing Specimens

**Figure 1:**
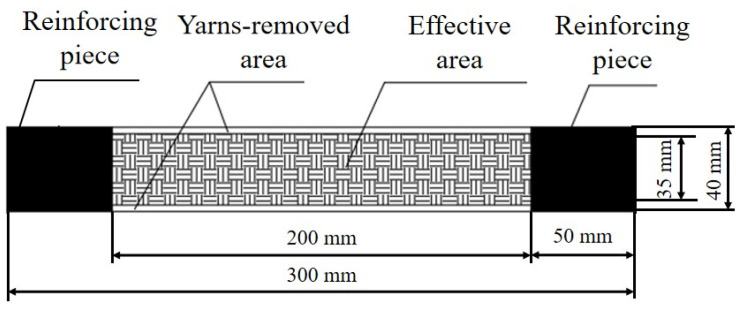
Schematic diagram of the specimen.

The specimen was designed according to standards ASTM D5035-200637 and GB/T 3923.1-201338 [43,44]. Specimens of 300 mm length and 40 mm width were cut along the warp direction from the original fabric. To minimize the effects of boundary yarns, the outermost several warp yarns were removed, forming yarn-removed areas on both sides in the loading direction. The final effective area of the specimen was 200 mm in length and 35 mm in width, as shown in Figure 1. To ensure load transmission and prevent premature failure due to stress concentration, glass fiber-reinforced epoxy composite tabs were bonded to both ends, curing for over 24 h to achieve sufficient interfacial strength.

#### 2.2.2. Quasi-Static Tensile Test Setup

**Figure 2:**
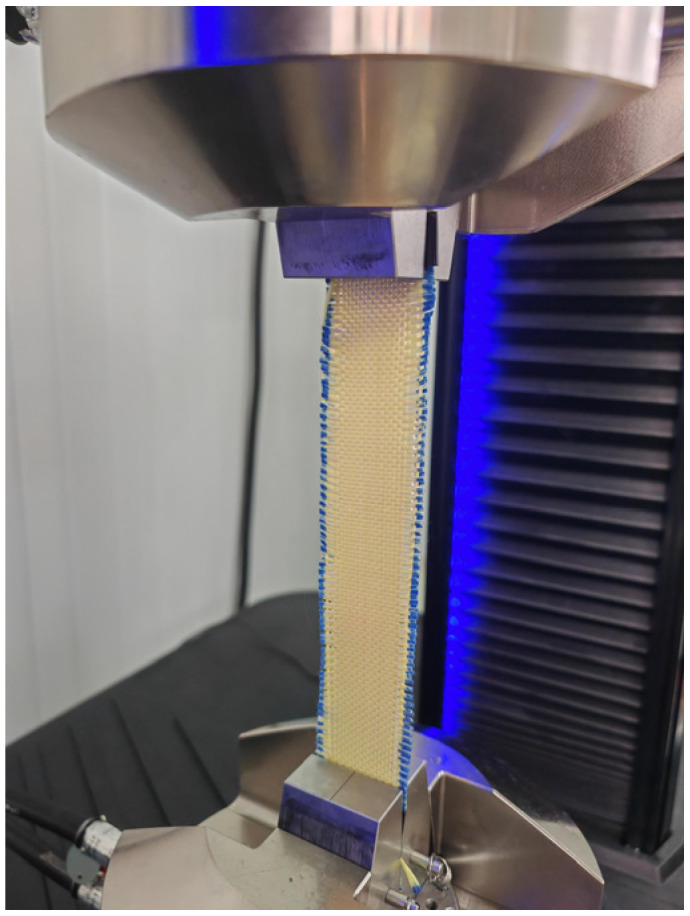
Quasi-static tensile test setup. (e.g., aramid fabric specimen).

A quasi-static tensile test was performed on a UTM-5105 servo-hydraulic testing machine with a maximum tensile force of 100 KN. Specimens were aligned along the axis of the machine and pre-stretched to ensure natural tautness, as shown in Figure 2. A constant crosshead speed of 20 mm/min was applied, corresponding to the requirement of the standard for fabrics with <8% elongation at break of 200 mm gauge length. The tensile loading and extension of the specimen were recorded during the testing process. The stress was obtained by dividing the tensile loading by the cross-sectional area of the specimen. The cross-sectional area is the cross-sectional area of the yarn times the number of yarns. A total of 30 tests were conducted for aramid, carbon, and UHMWPE fabrics, with five replicates along both warp and weft directions. Two aramid fabric specimens were excluded due to observed slippage in the clamping regions attributed to manufacturing defects, yielding 28 valid test results for subsequent analysis.

### 2.3. Dynamic Tensile Test

#### 2.3.1. Preparation of Dynamic Tensile Testing Specimen and Clamping Tool

**Figure 3:**
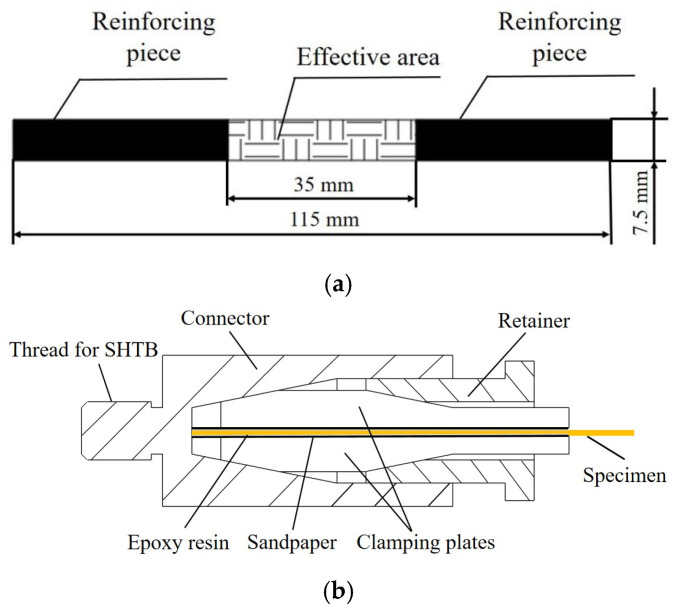
Design of the specimen and clamping tool; (**a**) schematic diagram of the specimen, (**b**) clamping tool of the specimen.

The design of the specimen was referenced to the methodologies of Shim [15] and Tan [45], featuring 115 mm total length with an effective area of 35 mm length and 7.5 mm width, as shown in Figure 3a. To increase the friction and avoid the slippage between the specimen and clamping tool, two 40 mm × 7.5 mm sandpapers were bonded at both ends. Epoxy resin was used to glue the specimen and sandpapers. The specimen obviously differed from the quasi-static one in having a shorter and narrower effective area. The design was primarily based on two considerations that the specimen with shorter length prevents untimely superposition of reflected and incident stress waves, ensuring temporal isolation of wave signals for accurate analysis. Additionally, the specimen with a narrower width promoted more uniform strain distribution across the surface of the specimen, enhancing the reliability of the test results.

For typical metal or polymer material, threading could be machined at both ends to facilitate attachment to the incident and transmission bars of the SHTB (Split Hopkinson Tensile Bar) device. However, a special load-transfer mechanism of fabric materials was exhibited due to the woven architecture, where the crossover points between warp and weft yarns serve as primary load-bearing nodes. Therefore, the fabric material was susceptible to damage under excessive clamping force and exhibited more sensitivity to stress concentration. Furthermore, inadequate clamping force may also cause slippage between the specimen and clamping tool, decreasing the accuracy of the results. Hence, an appropriate clamping tool is indispensable for establishing a stable connection between the specimen and the incident and transmission bars. To mitigate the effects of impedance mismatch, the clamping tool was made of the same material as the bars [45]. The clamping tool comprised two pairs of clamping plates, one pair of retainers, and one pair of connectors, with conical cavities featured in the retainer and connector was illustrated in Figure 3b. Firstly, the specimen is clamped between two clamping plates, which were then inserted into the connector. Subsequently, the retainer is screwed onto the threads of the connector. Owing to the conical surface of the clamping plate, as the retainer was tightened, the conical cavities of the connector glide along the surface, exerting pressure and clamping securely.

#### 2.3.2. Dynamic Tensile Testing Device

**Figure 4:**
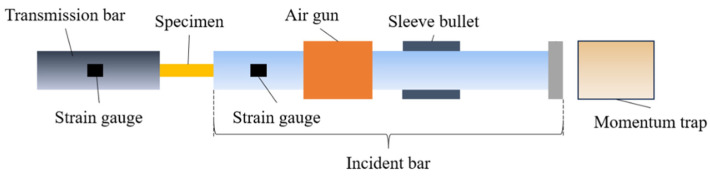
Schematic of the SHTB testing device.

The dynamic mechanical properties of fabrics were obtained through the SHTB device, as illustrated in Figure 4. Both the incident and transmission bars were made of aluminum alloy with a diameter of 20 mm, an elastic modulus of 72 GPa, a density of 2.8 × 10^3^ kg/m^3^, and a longitudinal wave velocity of 5070 m/s. The velocity of the sleeve bullet could be adjusted by varying the pressure of the gas chamber (0.4–1.2 MPa), resulting in a series of strain rates ranging from 2.0 × 10^2^ s^−1^ to 1.3 × 10^3^ s^−1^. The incident bar was impacted by a compressed air-driven sleeve bullet, generating a stress wave that propagates through the bar. To ensure that the specimen was loaded only by a single pulse, a momentum trap was adapted to the incident bar. Upon reaching the specimen, the stress wave was split into two parts that one part reflected back as the reflected wave, carrying strain-rate information, while the other part transmitted through the specimen as the transmitted wave for stress analysis. Strain gauges were used to measure the stress wave, with resistive gauges mounted on the incident bar and semiconductor gauges attached to the transmission bar. These sensors captured deformation signals, which were then amplified by a hyperdynamic amplifier and input into a data processing system for calculation, analyzing the incident, reflected, and transmitted wave components for dynamic analysis.

**Figure 5:**
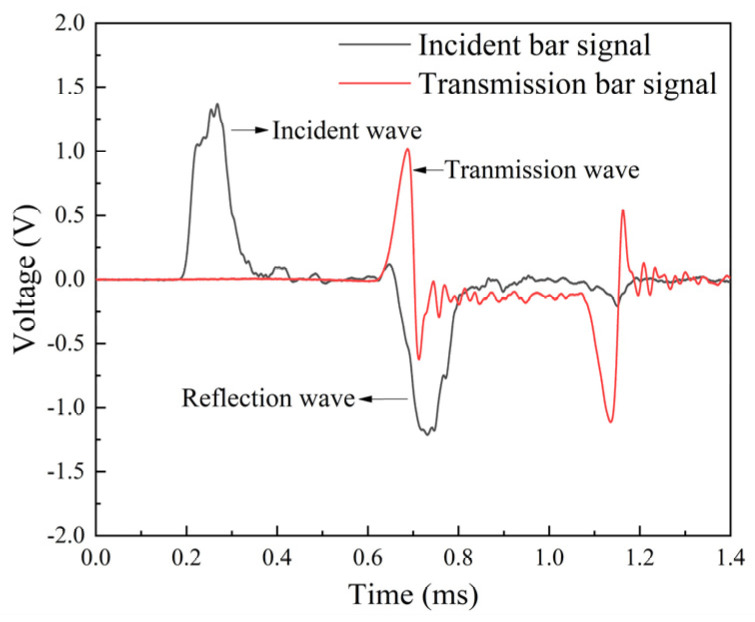
Typical strain waves in the SHTB test.

The typical voltage signals from the incident and transmission bars during the testing process are shown in Figure 5. The transmitted wave signal directly correlates with the stress evolution of the specimen, and the reflected wave is related to strain and strain rate. These measurements satisfy two fundamental assumptions: one-dimensional elastic wave propagation in the bars and uniform axial stress distribution in the specimen. Based on the basic assumptions, applying one-dimensional stress wave theory, the stress (σ), strain (ε), and strain rate ($\overset{•}{\varepsilon }$) of the specimen are derived from the processed voltage signals according to the equations developed by Kolsky [46]:

(1) $$\sigma (t)=\frac{A_{0}}{A_{S}}E_{0}\varepsilon_{t}(t)$$

(2) $$\overset{•}{\varepsilon }(t)=-\frac{2C_{0}}{L_{s}}\varepsilon_{r}(t)$$

(3) $$\varepsilon (t)=-\frac{2C_{0}}{L_{S}}\int_{0}^{t}\varepsilon_{r}(t)dt$$

(4) $$C_{0}=\sqrt{\frac{E_{0}}{\rho_{0}}}$$

where *C*_0_ is the elastic longitudinal wave velocity, ε is the strain of the specimen, *L*_s_ is the length of the specimen, ε*_r_* is the reflection strain, $\overset{•}{\varepsilon }$ is the strain rate of the specimen, σ is the stress of the specimen, ε*_t_* is the transmission strain, *A*_s_ is the cross-sectional area of the specimen, and *E*_0_ and *A*_0_ are the Young’s modulus and cross-sectional area of the bar.

### 2.4. Blast Test

#### 2.4.1. Preparation of Blast Testing Specimens

**Figure 6:**
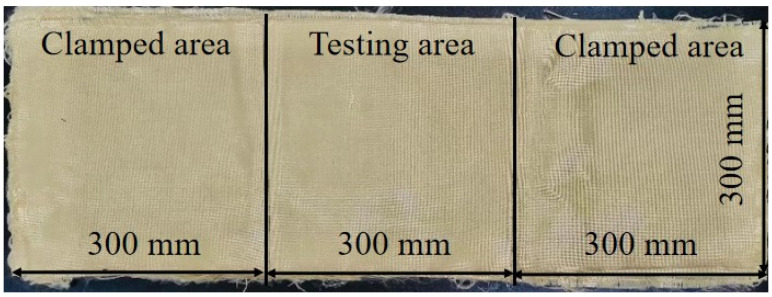
Size of the specimen (e.g., aramid fabric specimen).

**Figure 7:**
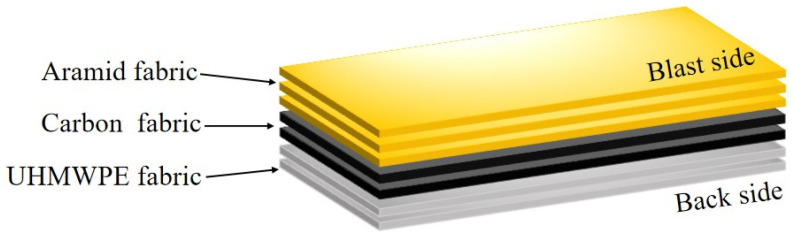
Laying sequence of a hybrid multi-ply fabric specimen (e.g., 3 mm thickness).

For instance, an aramid fabric specimen was prepared by cutting into dimensions of 900 × 300 mm^2^, featuring a testing area of 300 × 300 mm^2^ and two clamped areas of 300 × 300 mm^2^ on both sides, as illustrated in Figure 6. As an exploration, a “hybrid multi-ply fabric” configuration was developed, through stacking aramid, carbon, and UHMWPE fabrics with a thickness ratio of 1:1:1 to investigate synergistic blast-resistance mechanisms. For example, as a 3 mm thickness of hybrid multi-ply fabric specimen, there are 3 layers of aramid fabric, 2 layers of carbon fabric, and 2 layers of UHMWPE fabric stacked from the blast side to the back side, as shown in Figure 7. Stitching was applied along the boundary of the testing area to facilitate accurate control of fabric size and positioning during testing and clamping. The specimens were designed in three thickness specifications, including 3, 6, and 9 mm.

**Figure 8:**
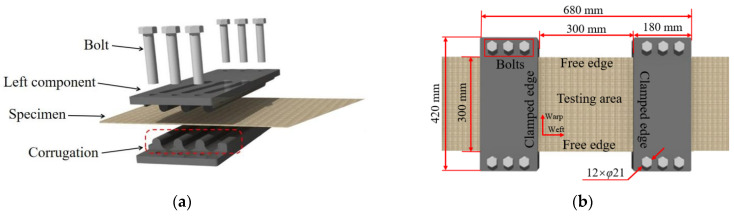
The fixture of the specimen; (**a**) side view, (**b**) front view.

To prevent the specimen from dislodging from the fixture subject to the blast loading, a fixture was designed specially, as shown in Figure 8. The fixture mainly consists of two different parts, which are respectively the left and the right components. Each part is composed of two steel plates, with corrugation at the contact points. The corrugation enhances the frictional forces between the fixture and the specimen (Figure 8a). Twelve high-strength bolts are employed to fasten the fixture. It is evident that the ends of the weft yarns are constrained, while those of the warp yarns remain free (Figure 8b). As a result, the weft yarns bear the majority of the external force, and the warp yarns supply the frictional force under the blast loading. Additionally, the fixture is employed because the axial length of the anti-explosion fabric bag exceeds the circumferential, making the circumferential force the dominant factor influencing the failure mode of the fabric.

#### 2.4.2. Blast Test Setup

**Figure 9:**
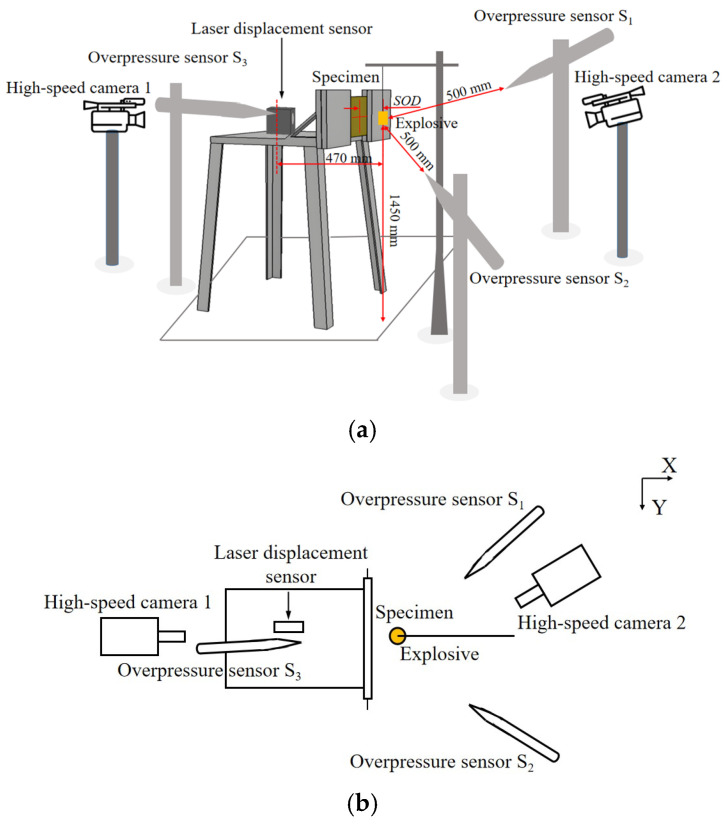
Schematic diagram of the test setup; (**a**) side view of the test setup, (**b**) top view of the test setup.

**Figure 10:**
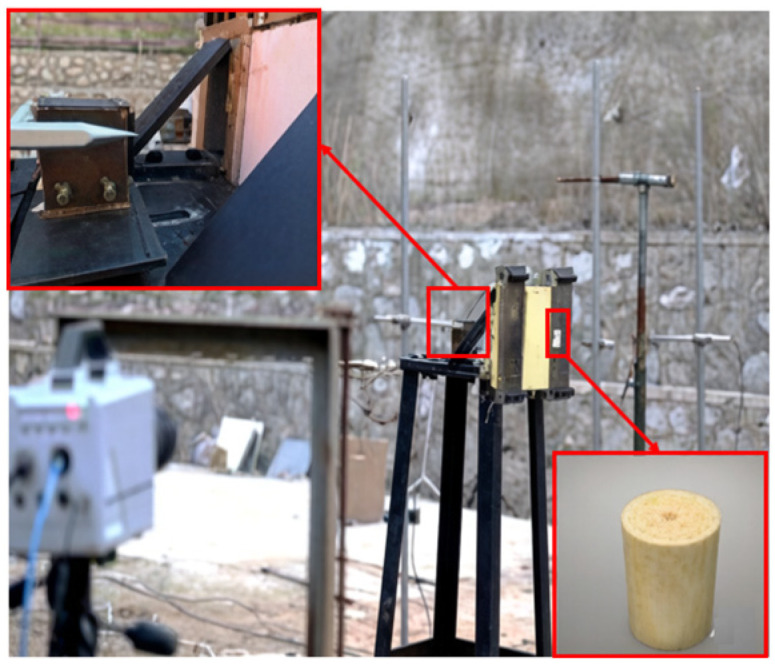
The photo of the test setup.

The test setup includes a specimen, a fixture, a TNT charge, two high-speed cameras, and three overpressure sensors, as shown in Figure 9 and Figure 10. To mitigate the influence of the reflected wave from the ground on the results, the fixture was vertically fixed, and the specimen was positioned 1450 mm away from the ground. A cylindrical explosive was suspended in front of the specimen, and the equivalent mass was used in four variants, respectively 10, 20, 60, and 100 g, with a density of 1.6 g/cm^3^. The type of overpressure sensors is PCB137B24B, and the range of measurement is 0–1725 KPa, which were designated as S_1_, S_2_, and S_3_. S_1_ and S_2_ were placed 500 mm from the center of the explosive on the blast side, and S_3_ was set 470 mm from the explosive on the back side. These sensors were maintained at the same horizontal height and aligned towards the explosive. The displacement of the central point of the specimen was measured through a laser displacement sensor, specifically the MTII LTS-300–200 type with a range of ±125 mm. Two Phtron SA5 high-speed cameras were employed to capture the dynamic response of the specimen during the explosion, with a frame rate set at 10,000 fps.

#### 2.4.3. Blast Test Conditions

A total of 13 test conditions were conducted, divided into 4 groups as shown in Table 2. Firstly, single-material fabrics were tested under the same TNT charge mass (60 g) and standoff distance (100 mm). Additionally, specimen identification follows a systematic method. The alphabet corresponds to the fabric materials: ‘A’ denotes aramid fabric, ‘C’ represents carbon fabric, and ‘U’ indicates UHMWPE fabric. The hybrid multi-ply fabric, designated as ‘H’, follows an A-C-U stacking sequence from blast side to back side. For instance, ‘A6-1’ refers to aramid fabric with a thickness of 6 mm and ranked ‘1’ in the blast test of fabric of single-material, ‘H3-6’ refers to hybrid multi-ply fabric with a thickness of 3 mm and ranked ‘6’ in the blast test of fabric material of hybrid type.

## 3. Results and Discussion

### 3.1. Quasi-Static Tensile Behavior and Properties of Fabrics

**Figure 11:**
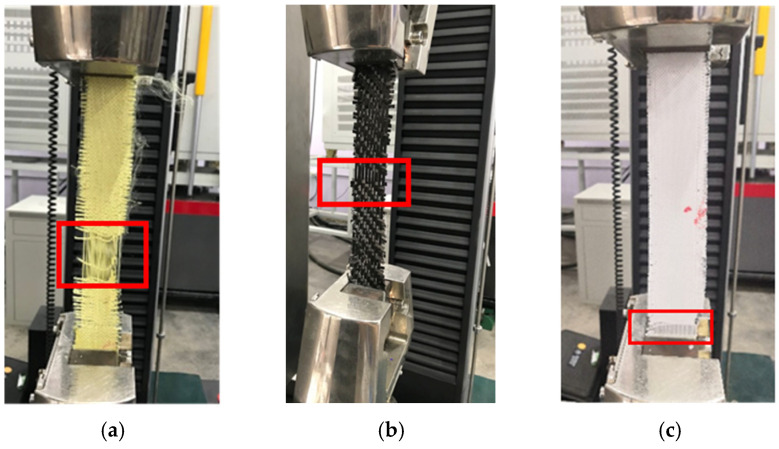
Failure modes of the specimens under the quasi-static tensile test; (**a**) Aramid fabric, (**b**) Carbon fabric, (**c**) UHMWPE fabric.

The failure modes of the specimens under the quasi-static tensile test are compared in Figure 11. Aramid fabric showed progressive failure initiation at the yarn interlacing points or inherent weak spots, leading to progressive yarn breakage and fibrillation. Conversely, carbon fabric experienced brittle failure, exhibiting no visible carbon fiber pull-out or fuzzing at the breakage location. It was indicated that the high stiffness and breakage occurred with minimal plastic deformation. For UHMWPE fabric, the smooth surface made it particularly susceptible to stress concentration and localized failure due to the clamping force. The fibrillation and yarn breakage were also observed in the UHMWPE fabric, which indicated considerable ductility. Despite these different damage morphologies, the fabric yarns slipped in the transverse direction, and the longitudinal yarns were gradually elongated but still not completely broken.

**Figure 12:**
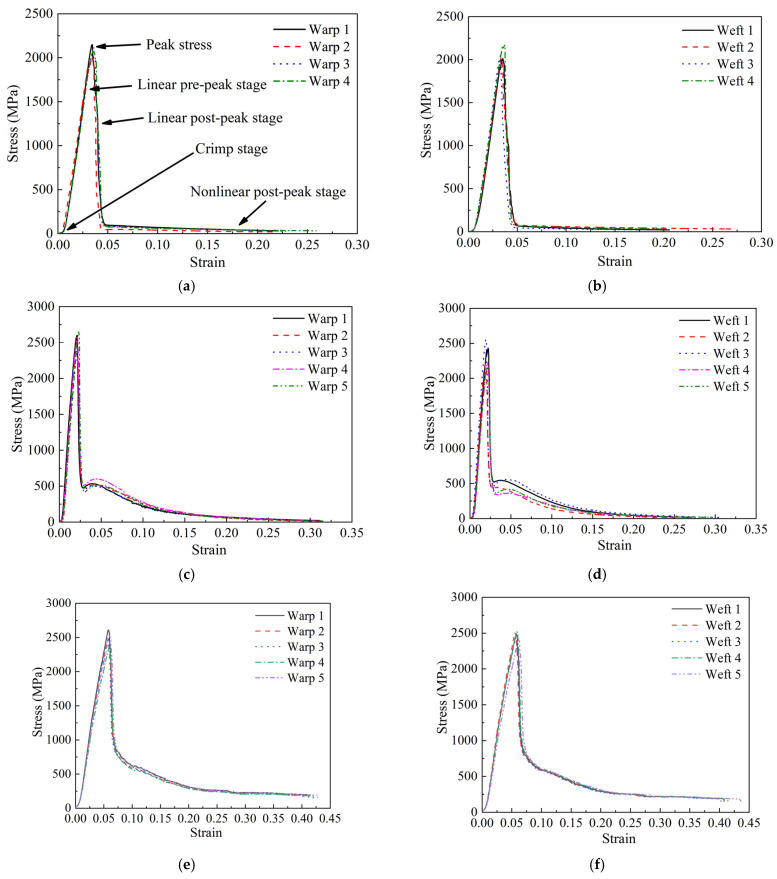
Stress–strain curves in warp and weft directions of specimens under quasi-static tensile test; (**a**) Stress–strain curves of aramid fabric in warp direction, (**b**) Stress–strain curves of aramid fabric in weft direction, (**c**) Stress–strain curves of carbon fabric in warp direction, (**d**) Stress–strain curves of carbon fabric in weft direction, (**e**) Stress–strain curves of UHMWPE fabric in warp direction, (**f**) Stress–strain curves of UHMWPE fabric in weft direction.

As mentioned in Section 2.2.2, at least four quasi-static tensile tests in the warp and weft directions of each fabric were conducted. The repeatability of the test results for the three fabrics was satisfied, indicating the validity of the test, as shown in Figure 12. Additionally, during the weaving process, particularly on projectile looms, warp and weft yarns are subjected to considerable tension. Frictional wear also occurs as the warp yarns pass through the reed and during the high-impact beat-up of the weft. These actions degrade the tensile properties of the yarns and could lead to a measurable difference in strength between warp and weft. Consequently, both fabrics of warp and weft directions were tested in this study. Interestingly, the tensile properties of the warp and weft specimens were nearly identical, indicating that weaving caused minimal damage to the fabric in both directions, as shown in Figure 12.

**Figure 13:**
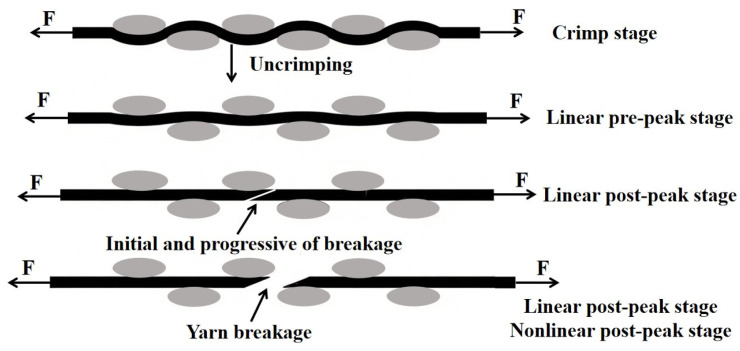
Schematic of the quasi-static tensile response of a fabric specimen.

Figure 13 is a schematic of stress–strain response during the test, corresponding to the four distinct stages in Figure 12a. These gray ovals represent the cross-section of the individual yarns. Their positions are perpendicular to the loading direction of the stretched yarn. The response included the crimp stage, linear pre-peak stage, linear post-peak stage, and nonlinear post-peak stage. In the first stage, the stress–strain curve showed a relatively large increase in strain at a low stress level. It was associated that the small force required to straighten the inherent crimp in woven fabrics was referred to as the crimp stage. Once the straightened yarns started to take more loads, the slope of the curve increased, and this region was referred to as the linear pre-peak stage. After reaching the peak stress, the stress decreased rapidly due to the initial and progressive breakage of the yarns, which was regarded as the linear post-peak stage. When the stress dropped to a lower value, the decrease slowed down and lasted for a long time until the stress value reached zero. At this point, most of the yarns had already broken, but the specimen did not fail completely. This stage was considered the nonlinear post-peak stage.

**Figure 14:**
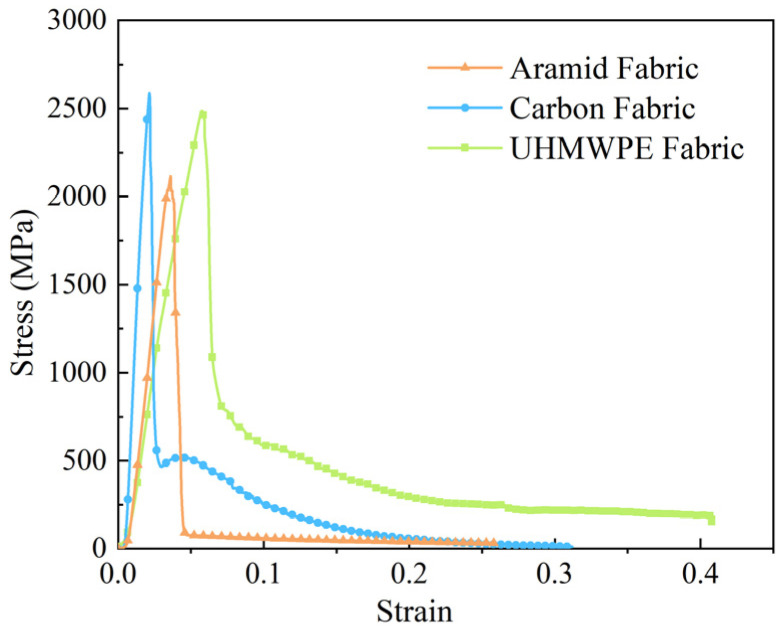
Typical stress–strain curves of fabrics under quasi-static tensile test.

Based on the test results, to focus on the properties of fabric materials under quasi-static tensile, the average of warp and weft material response was extracted, and typical stress–strain curves characterizing the constitutive relationships of the three fabric materials were obtained, as shown in Figure 14. It was found that the stress–strain curve of carbon fabric reached peak stress at a strain of approximately 0.02 followed by an abrupt, near-vertical stress drop. It was indicated that carbon fabric had high modulus, strength, but limited ductility. Conversely, the stress–strain curve of UHMWPE fabric reached peak stress at a strain of approximately 0.06. And overall response was marked by an extended stress plateau, signifying a progressive breakage process that facilitated energy absorption over a wide strain range. It reflected that high ductility and toughness of UHMWPE fabric. The aramid fabric presented an intermediate quasi-static tensile behavior. While also exhibiting a sharp linear post-peak decline, the stress decrease was more gradually than that of carbon fabric, suggesting also a progressive breakage mechanism.

**Figure 15:**
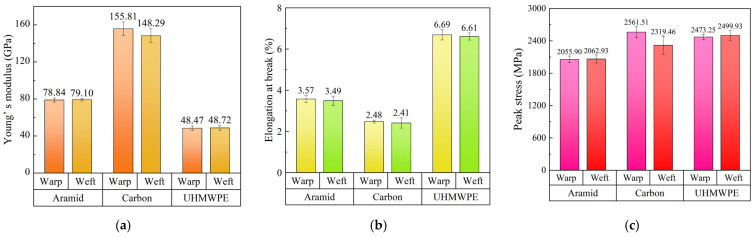
Quasi-static tensile properties of fabrics in warp and weft directions; (**a**) Young’ s modulus, (**b**) elongation at break, (**c**) peak stress.

The average test results and standard deviation for fabrics are shown in Figure 15. Young’s modulus is the slope of the linear pre-peak stage. Peak stress is the maximum stress at the end of the linear pre-peak stage. The ratio of the maximum length that a fabric could extend before breakage to the original length is the elongation at break. The maximum length refers to the length of the specimen at the end of the third stage. Therefore, the strain at the end of the linear post-peak stage is elongation at break. It was found that the tensile properties of the warp and weft directions of individual fabrics are similar. From the perspective of material comparison, the Young’s modulus of carbon fabric was higher than that of aramid and UHMWPE fabrics under a quasi-static tensile test. It was indicated that carbon fabric had the highest stiffness. However, compared to the other two fabrics, carbon fabric had the smallest elongation at break. The maximum elongation at break of UHMWPE fabric indicated excellent ductility. In addition, the peak stress of carbon and UHMWPE fabrics was relatively close, slightly higher than that of the aramid fabric. An intermediate level of quasi-static tensile properties of aramid fabric was observed, demonstrating better-balanced stiffness and toughness.

### 3.2. Dynamic Tensile Behavior and Properties of Fabrics

**Figure 16:**
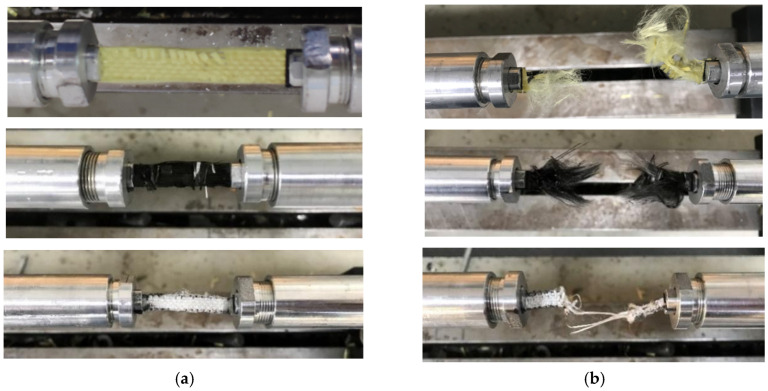
Failure modes of the specimens under the dynamic tensile test; (**a**) before the dynamic tensile test, (**b**) after the dynamic tensile test.

The failure modes of fabric specimens under the dynamic tensile test are compared in Figure 16. Similar to the results of the quasi-static tensile test, distinct failure modes were observed. For aramid and carbon fabrics, complete failure occurred within the effective gauge area in the middle position. The filaments of aramid fabric became fluffy along the loading direction, while the carbon fabric yarns showed brittle breakage, characterized by extensive fiber pull-out without fluffy. In contrast, the UHMWPE fabric specimen did not completely fail. And the breakage position was highly localized. It may be attributed to the substantial plastic deformation of the UHMWPE fabric, which reduced the damage and concentrated the breakage process within the effective area.

**Figure 17:**
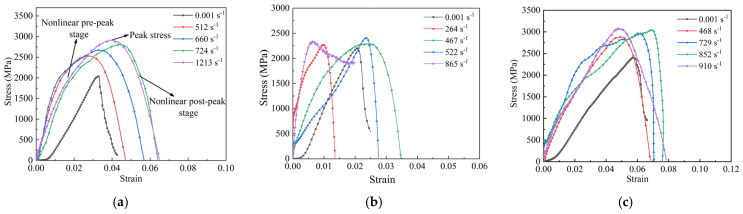
Stress–strain curves of fabrics at different strain rates; (**a**) aramid fabric, (**b**) carbon fabric, (**c**) UHMWPE fabric.

The stress–strain curves of fabrics at different strain rates are shown in Figure 17. Two stages of the dynamic tensile response are shown in Figure 17a, including the nonlinear pre-peak stage and the nonlinear post-peak stage. Within the range of strain rates investigated, the stress–strain responses of all fabrics lacked a crimp stage and a nonlinear post-peak stage. Once the yarns took more loads, the slope of the curve increased nonlinearly, and this region was referred to as the nonlinear pre-peak stage. The nonlinear post-peak stage was marked by a stress decrease after the peak stress, corresponding to yarn breakage. Consequently, once individual fibers reached the peak stress, the stress could not be redistributed effectively to neighboring fibers. It was found that the localized overloading and abrupt breakage across the yarns occurred shortly after the peak stress was reached. As a result, the nonlinear post-peak stage of the stress–strain curve was not observed. And the response was nearly vertical stress drop from the peak stress to final failure, characteristic of dynamic brittle failure.

**Figure 18:**
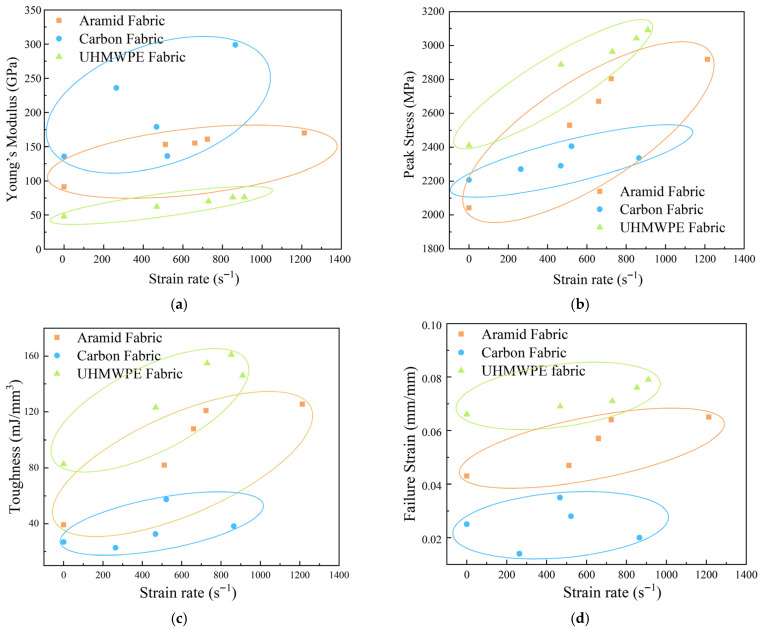
Variation in material properties with strain rates under the dynamic tensile test; (**a**) Young’s modulus, (**b**) peak stress, (**c**) toughness, (**d**) failure strain.

The material properties for fabrics at different strain rates are plotted and compared in Figure 18. Toughness is obtained by integrating the stress–strain curve, which represents the energy absorbed by the fabric before failure. The strain when the stress dropped to approximately zero is the failure strain. Young’s modulus of the fabrics is measured by fitting the stress rise stage linearly. It was found that the Young’s modulus, peak stress, toughness, and failure strain of aramid and UHMWPE fabrics increased with the strain rate, exhibiting typical strain-rate sensitivity. However, similar properties of carbon fabric were shown across varying strain rates, demonstrating strain-rate insensitivity in the dynamic response. The Young’s modulus of carbon fabric was generally higher than that of aramid and UHMWPE fabrics, while the peak stress, toughness, and failure strain of UHMWPE fabric were higher than those of aramid and carbon fabrics under the strain rates investigated. Despite the high peak stress, the carbon fabric possessed the lowest failure strain, resulting in the smallest area under the stress–strain curve. It was indicated that a limited capacity for energy absorption prior to failure was a key reason for brittle failure. On the contrary, UHMWPE fabric expressed better strength and ductility at high strain rates.

### 3.3. Blast Resistance Performance of Fabrics Under Blast Loading

#### 3.3.1. Calculation of Wave Impedance for Fabrics

At the moment of explosion, the shock wave energy generated by the TNT explosive was mainly transmitted in the form of stress waves. When the stress waves were transmitted to the air-fabric interface, the wave of transmission and reflection would occur. In order to elucidate the dynamic response and failure modes of the aramid, carbon, and UHMWPE fabrics under the blast loading from the perspective of stress wave propagation, this section will present a theoretical analysis based on wave impedance. Wave impedance (*Z*) is defined as the product of yarn bulk density (*ρ*) and the propagation speed of stress wave in the medium (*C*). A higher wave impedance signified a greater capacity for reflecting energy of the incident shock wave at the surface, thereby reducing the energy transmitted into the fabric material. The wave impedance (*Z*) and wave speed (*C*) of the specimen are derived according to the equations:

(5) Z=ρ×C

(6) $$C=\sqrt{\frac{E_{d}}{\rho }}$$

where ρ is the yarn bulk density from Table 1, C denotes the propagation speed of stress wave in the medium, and E_d_ is the Young’s modulus of fabric at the maximum strain rate investigated.

The parameters related to wave impedance for fabrics are shown in Table 3. The results showed that there were differences in the wave impedance of the fabrics, with carbon fabric being the highest, followed by aramid fabric, and then UHMWPE fabric. The wave impedance of fabric was the primary factor determining the distribution of shock wave energy at the interface, while the dynamic response and failure modes also depended on how the fabric dissipated the transmitted energy according to the properties of the fabric material. From this perspective, the highest wave impedance of the carbon fabric resulted in the reflection of a substantial portion of the incident shock wave energy on the blast side. The reflection mechanism generated highly localized compressive stress. In contrast, the lowest wave impedance of the UHMWPE fabric allowed for greater transmission of the shock wave into the inner layers. The aramid fabric offered a balanced response that reflected a portion of the shock wave to attenuate loading with intermediate wave impedance while simultaneously dissipating energy through yarn fibrillation and inter-yarn slippage.

#### 3.3.2. Dynamic Response of Single-Material and Hybrid Multi-Ply Fabrics

**Figure 19:**
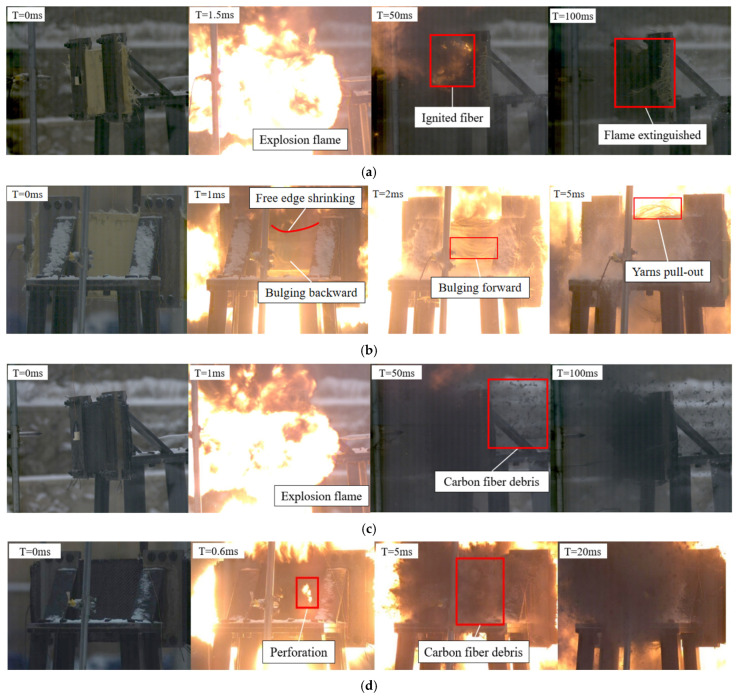

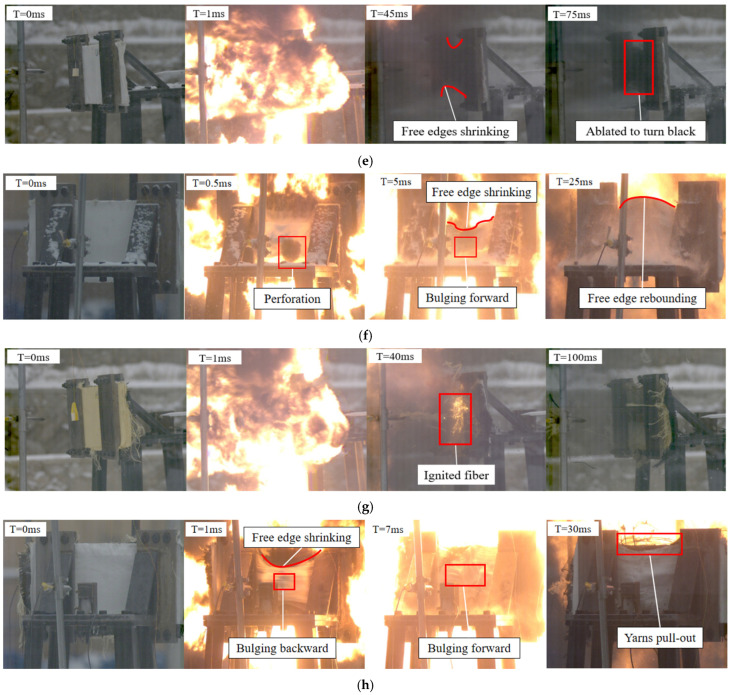
Dynamic response of fabrics in tests 1 (A6-1), 2 (C6-1), 3 (U6-1), and 10 (H6-1); (**a**) blast side of test 1 (A6-1), (**b**) back side of test 1 (A6-1), (**c**) blast side of test 2 (C6-1), (**d**) back side of test 2 (C6-1), (**e**) blast side of test 3 (U6-1), (**f**) back side of test 3 (U6-1), (**g**) blast side of test 10 (H6-1), (**h**) back side of test 10 (H6-1).

The dynamic response of the fabrics in tests 1, 2, 3, and 10 is compared and studied as shown in Figure 19. And ‘T = 0 ms’, which corresponded to the immediate moment preceding detonation. In test 2, it was observed that the central region of the carbon fabric showed brittle failure at approximately 5 ms post-detonation, resulting in carbon debris ejected in a cloud-like manner towards the back side, as shown in Figure 19c,d. This indicated that carbon fabric could not dissipate energy through large deformation due to the low toughness and failure strain. Additionally, carbon fabric generated localized stress far exceeding the dynamic peak stress, leading to widespread, instantaneous yarn breakage. On the contrary, UHMWPE fabric demonstrated a markedly different dynamic response dominated by plastic deformation in test 3. The UHMWPE fabric exhibited in-plane deformation and vibration upon shock wave due to low Young’s modulus and high toughness, as shown in Figure 19e,f. It was shown that a substantial portion of the energy was dissipated through deformation. Furthermore, black smoke was observed at 0.5 ms, indicating that the UHMWPE fabric exhibited perforation failure. The aramid fabric bulged backward, subject to the blast loading on the central region at 1 ms in test 1. After the positive pressure duration from the shock wave, the aramid fabric began to rebound and bulge forward under the combined effects of the negative pressure wave and the accumulated elastic potential energy, as shown in Figure 19a,b. In addition, no perforation occurred in the aramid fabric compared with UHMWPE and carbon fabrics because of more balanced stiffness and toughness. Due to the influence of the boundary conditions imposed by the clamping tools, the warp yarns experienced frictional slip, causing the yarns to pull out. Therefore, the brittle failure of carbon fabric may correlate with minimal toughness and failure strain, whereas the other two fabrics could reduce the severity of failure through the deformation (mainly including the deformation energy of yarns and the energy absorption due to inter-yarn slippage) and kinetic energy generated by vibration. In test 10, the in-plane deformation, vibration, and yarn pull-out were observed on the hybrid multi-ply fabric specimen without perforation, as shown in Figure 19g,h.

**Figure 20:**
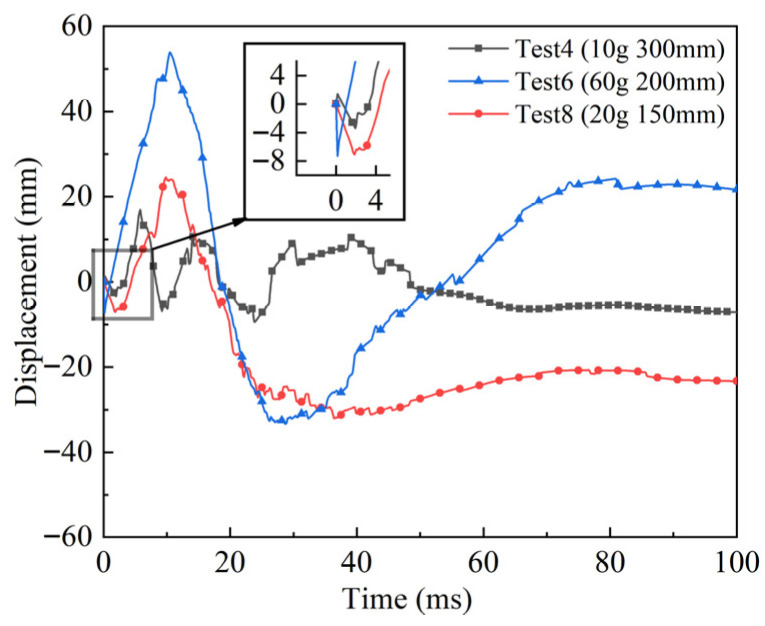
Displacement-time history curves of tests 4, 6, and 8.

As mentioned in Section 2.4.2, a laser displacement sensor was used to record the deformation process of the hybrid multi-ply fabric without perforation failure during the explosion. The measurement point was the center of the fabric on the back side. The equilibrium position of the fabric specimen before the explosion was recorded as the zero-displacement point, and the laser sensor was set to have a negative displacement when the fabric bulged towards the sensor direction and a positive displacement when it bulged towards the explosive direction. The displacement-time history curves of tests 4, 6, and 8 are compared in Figure 20. The scaled distances for tests 4, 6, and 8 are 1.39, 0.51, and 0.55 kg/m^1/3^, respectively. The inset illustrated that the hybrid multi-ply fabrics firstly bulged toward the sensor (negative displacement), during the initial 0–2 ms. The peak negative displacements in tests 6 and 8 were notably larger and closer in magnitude compared to test 4, with test 6 exhibiting a higher initial velocity. It was indicated that the initial response was governed primarily by the high wave impedance aramid and carbon fabric layers, whose wave-reflection behavior induced the bulge toward the sensor. After approximately 2 ms, the hybrid multi-ply fabric bulged toward the explosive (positive displacement). The maximum positive displacements in tests 6, 8, and 4 were 53.6 mm, 23.9 mm, and 17.0 mm, respectively. This stage was driven by the explosive negative-pressure phase and the stored elastic potential energy of the hybrid multi-ply fabric. Consequently, the UHMWPE fabric layers on the back side underwent extensive stretching upon receiving the load transferred through the stitched boundaries. Due to the highest loading input in test 6, the plastic deformation of the UHMWPE fabric was the most fully developed, leading to the largest positive displacement. Following the positive peak displacement, the displacement-time curves oscillated and gradually stabilized. The final residual displacements for tests 4, 6, and 8 were −6.9 mm, +22.3 mm, and −23.3 mm, respectively. A clear distinction was observed in the final deformation direction between tests 6 and 8. It may be attributed that the hybrid multi-ply fabric in test 6 possessed sufficient kinetic energy to enter a stage of plastic deformation dominated by the UHMWPE fabric layers compared with test 8. As a result, the hybrid multi-ply fabric in test 6 showed nearly a complete oscillation cycle. In contrast, the limited loading in test 8 provided inadequate kinetic energy for the hybrid multi-ply fabric to return to its equilibrium position after the initial negative rebound. The hybrid multi-ply fabric subjected to the lowest loading in test 4, responded essentially elastically and therefore returned to the original equilibrium position.

#### 3.3.3. Failure Modes of Single-Material and Hybrid Multi-Ply Fabrics

**Figure 21:**
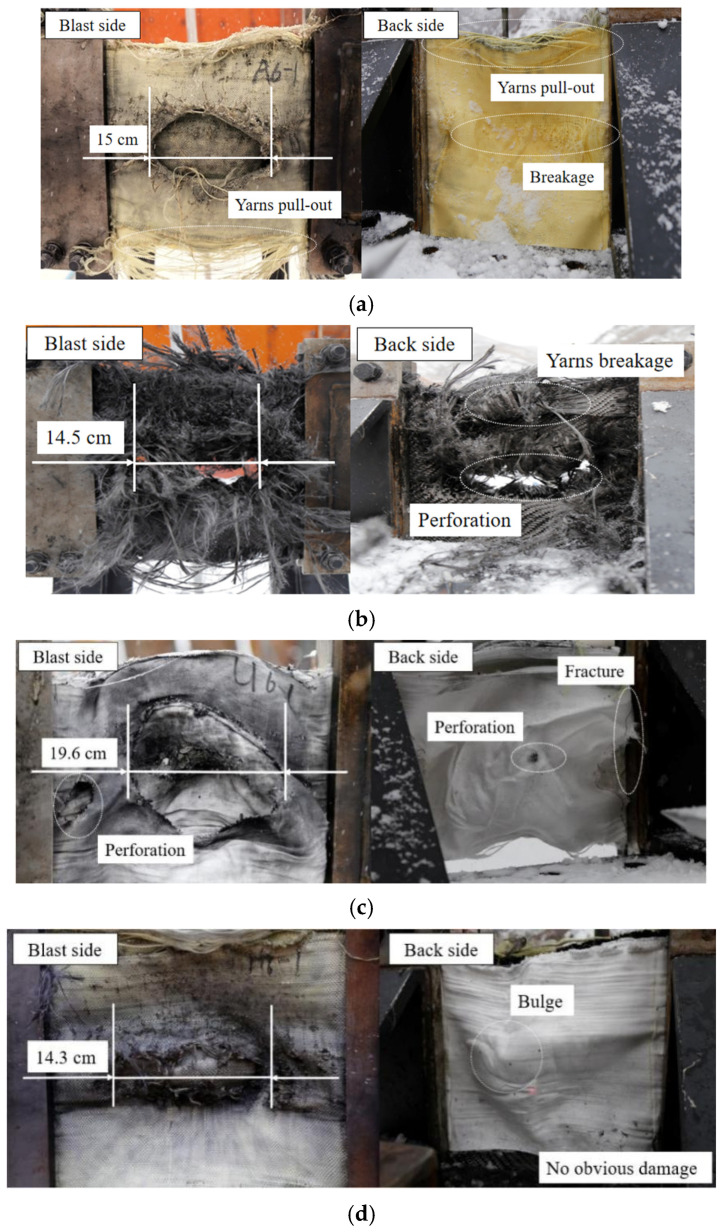
Failure modes of fabrics in tests 1 (A6-1), 2 (C6-1), 3 (U6-1), and 10 (H6-1); (**a**) test 1 (A6-1) 60 g 100 mm, (**b**) test 2 (C6-1) 60 g 100 mm, (**c**) test 3 (U6-1) 60 g 100 mm, (**d**) test 10 (H6-1) 60 g 100 mm.

The typical failure modes of fabrics could be summarized as follows. Under the studied conditions, there are mainly four kinds of failure modes:

(1) Perforation failure of partial layers (taking test 1 as an example, only the first layer where perforation occurred is shown in Figure 21a).
(2) Perforation failure (taking test 2 as an example, all layers where perforation occurred are shown in Figure 21b).
(3) Fracture (including tear failure within the in-plane and the boundary).
(4) Yarn breakage or slippage.

The failure modes of fabrics in tests 1 (A6-1), 2 (C6-1), 3 (U6-1), and 10 (H6-1) under the condition of 60 g TNT and 100 mm SoD could be summarized by analyzing the test results as shown in Figure 21. Perforation failure and yarn breakage were observed in the carbon fabric, with a maximum perforation length of 14.5 cm, as shown in Figure 21b. It may be attributed to the highest wave impedance of the carbon fabric, which resulted in the reflection of a greater portion of the incident shock wave on the blast side of the carbon fabric, thereby generating high localized compressive stress. Due to the air gaps between the fabric layers, when the transmitted stress wave propagates to the back of the fabric layer, it will be reflected back in the form of a reflected tensile wave due to the mismatch in wave impedance with the air. Additionally, the transmitted wave reflected on the front surface of the next layer of fabric. The new wave, when superimposed with the residual part of the original wave inside the fabric, resulted in a cumulative stress that exceeded the dynamic peak stress of the carbon fabric. The perforation failure of all layers in Figure 21b further demonstrated the rapid propagation and superposition of stress waves between layers. Due to the maximum Young’s modulus and low toughness of the carbon fabric, brittle yarn breakage occurred, manifested as perforation failure.

**Figure 22:**
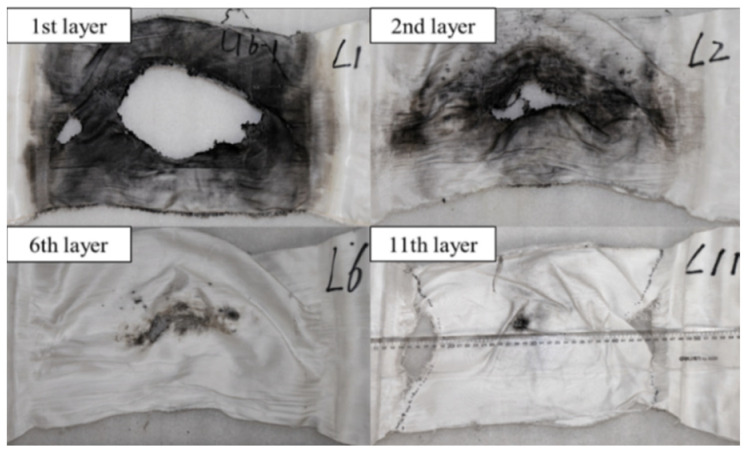
Perforation failure of representative layers of UHMWPE fabric in test 3 (U6-1).

Each reflection and transmission of the shock wave at the air-gap interface reduced the overpressure but prolonged the duration. It transformed a short, sharp shock pulse into a relatively smooth and sustained one, providing more favorable conditions for fabrics that dissipated energy through large deformation. Perforation failure was also observed on the UHMWPE fabric. The failure mechanism of UHMWPE fabric was linked to the lowest wave impedance and high toughness. Compared to carbon fabric, the lower wave impedance allowed a greater proportion of the incident shock wave to transmit into the UHMWPE fabric. Furthermore, the duration of the shock wave within the specimen was prolonged due to the lower wave speed of the UHMWPE fabric and reflection and transmission of the shock wave at the air-gap interface. Although the first layer of UHMWPE fabric exhibited the most severe perforation due to directly bearing the highest overpressure, with a maximum perforation length of approximately 19.6 cm, as shown in Figure 21c. As the duration of the shock wave increased, it took advantage of the high toughness of the UHMWPE fabric. The subsequent layers had sufficient time to experience plastic deformation through mechanisms such as fiber stretching and slipping, dissipating the remaining energy. Therefore, the severity of perforation failure in the UHMWPE fabric progressively diminished from the blast side to the back side due to energy attenuation, as illustrated in Figure 22. Additionally, the combined action of the reflected tensile wave and the stress concentration at the clamping region induced a fracture at the boundary on the back side.

In contrast to the perforation failure observed in the two other fabrics, only perforation failure of the first layer was observed on the aramid fabric with a perforation area approximately 15 cm in length, as shown in Figure 21a. Due to the reflected tensile wave, induced partial yarn breakage in the aramid fabric on the back side. Furthermore, yarn pull-out failure was observed at the free edges on both the blast and back sides of the aramid fabric layers. It may be attributed that the free edges represented the regions of weakest constraint for the yarns, where shear stress at inter-yarns could more readily induce yarn slippage. It confirmed that the aramid fabric dissipated a portion of the energy through localized inter-yarn friction and yarn breakage.

Under identical test conditions, the hybrid multi-ply fabric demonstrated the least failure. Perforation failure occurred on the first layer, with a maximum length of approximately 14.3 cm. Additionally, the hybrid multi-ply fabric only bulged, with no obvious damage on the back side, as shown in Figure 21d. Three types of fabrics each have their advantages in a hybrid multi-ply fabric. The aramid fabric on the blast side leveraged comparatively high wave impedance. A portion of the incident shock wave was reflected by the aramid fabric, thereby reducing the shock wave transmitted into the inner fabric layers and decreasing the overpressure, protecting the carbon fabric in the middle layer. The shock wave induced in-plane stretching of the aramid fabric during the deformation. Energy was dissipated through both inter-yarn slippage and yarn breakage. In addition, the carbon fabric located in the middle position provided high in-plane stiffness to the assembly. It effectively homogenized the attenuated loading transmitted through the aramid fabric layers, preventing localized stress concentration. At this point, as the overpressure has been reduced, the risk of complete brittle failure was effectively controlled. At last, the remaining energy was dissipated through plastic deformation by the UHMWPE fabric, which was characterized by high toughness. The process effectively converted the residual kinetic energy into internal strain energy within the material. The configuration also confirmed the conclusion reached by Chen et al. [47], who conducted numerical and experimental studies on the impact resistance of aramid and UHMWPE fabric configurations and found that aramid fabric as the impact side and UHMWPE fabric as the back side resulted in superior impact resistance.

In this study, displacement-time history was not measured for all configurations. The experimental design prioritized the systematic comparison of failure modes and the effect of overpressure attenuation across four configurations (aramid, carbon, UHMWPE fabrics, and hybrid multi-ply fabric) under identical test conditions. Since the carbon and UHMWPE fabrics experienced perforation failure, their displacement of the center point would not represent the global structural deflection, making such measurements physically non-equivalent for direct comparison. Therefore, displacement-time data were only acquired for the non-perforated hybrid multi-ply fabric to investigate the dynamic response of an intact fabric specimen, respectively, as presented in Figure 20. Consequently, a direct comparison of peak deflection among the four configurations mentioned above was not performed.

Owing to the employment of different fabrics in different positions of the hybrid multi-ply fabric, multiple types of failure modes were exhibited on each fabric layer. In partial tests, the specimens had perforation failure, while in other tests, only the aramid fabric exhibited partial-layer perforation failure. In order to provide a clearer description, the perforation failure was described as ‘A(X) C(X) U(X)’, which was named the test conditions. A summary of the perforation failure observed in the hybrid multi-ply fabric specimens is shown in Table 4.

(1) Aramid fabric layers

**Figure 23:**
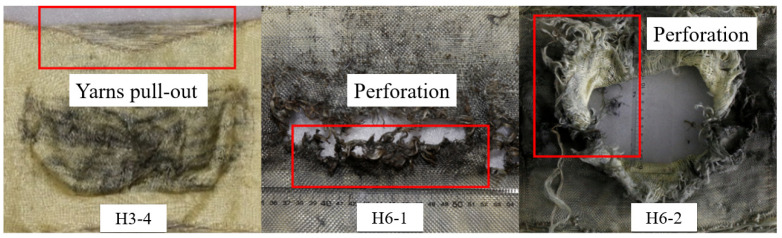
Failure modes of aramid fabric layers in tests 7 (H3-4), 10 (H6-1), and 11 (H6-2).

Serving as the blast side layer of the hybrid multi-ply fabric, the aramid fabric reflected a portion of the incident shock wave due to relatively high wave impedance. The reflection behavior effectively attenuated the peak pressure transmitted into the inner layers. Unlike the failure modes of carbon fabric, which exhibit brittle failure under blast loading, aramid fabrics exhibited superior energy absorption capacity through yarn breakage and slippage mechanisms. The various failure modes of aramid fabric layers in tests 7 (H3-4), 10 (H6-1), and 11 (H6-2) were summarized and compared in Figure 23. The shock wave energy was insufficient to induce yarn breakage on the aramid fabric in test 7, where the blast loading intensity was relatively low. Instead, energy dissipation occurred primarily through inter-yarn slippage and pull-out at the free edges. In contrast, the specimens were subjected to higher loading intensities in tests 10 and 11. Under these conditions, the elevated overpressure generated substantial local compressive stresses in the central region of the hybrid multi-ply fabric. Concurrently, the tensile stress wave reflected from the back side interacted with the incident compressive wave, creating a complex stress state that exceeded the dynamic peak stress of each layer of aramid fabric. The stress superposition caused instantaneous yarn breakage in the central area, ultimately leading to perforation failure.

(2) Carbon fabric layers

**Figure 24:**
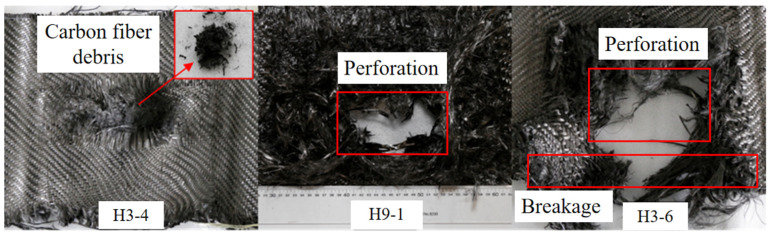
Failure modes of carbon fabric layers in tests 7 (H3-4), 12 (H9-1), and 9 (H3-6).

The shock wave attenuated by the aramid fabric layer was primarily experienced by the carbon fabric in the middle layers of the hybrid multi-ply fabric. Additionally, carbon fabric improved the bending stiffness of the hybrid multi-ply fabric through boundary stitching with the other two fabrics. The various failure modes of carbon fabric layers in tests 7 (H3-4), 12 (H9-1), and 9 (H3-6) are summarized in Figure 24. In test 7, after being attenuated as it traversed the aramid fabric layers, the low loading intensity combined with the high wave impedance of the carbon fabric generated localized compressive stress through surface wave reflection. The shock wave intensity only induced brittle breakage on the blast side of the carbon fabric, producing carbon fiber debris. In test 12, despite attenuation by air and aramid fabric, the peak pressure imposed on the carbon fabric remained sufficiently high due to the intense initial loading. The high pressure generated substantial compressive stresses on the surface, while simultaneously forming tensile waves reflected from the back side. Concurrently, under the influence of boundary conditions, the perforation failure occurred through the combined action of shock wave loading and tensile stresses induced by the global deformation. In test 9, due to the reduced number of layers, the failure mode of the carbon fabric layer became more severe. The yarns surrounding the perforation were subjected to tensile stresses far exceeding the dynamic peak stress, resulting in yarn breakage. Within the hybrid multi-ply fabric, the carbon fabric primarily functions as a sacrificial energy-absorbing layer through the brittle breakage mechanism.

(3) UHMWPE fabric layers

**Figure 25:**
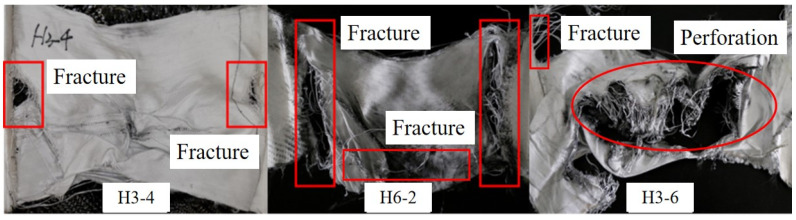
Failure modes of UHMWPE fabric layers in tests 7 (H3-4), 11 (H6-2), and 9 (H3-6).

The initial shock wave underwent multiple attenuation processes through the fabric layers, leading to a substantial reduction in the peak pressure experienced by the UHMWPE fabric on the back side. The failure modes of carbon fabric layers in tests 7 (H3-4), 11 (H6-2), and 9 (H3-6) are summarized and shown in Figure 25. In test 7, the attenuated energy was insufficient to induce perforation failure in the UHMWPE fabric. Owing to the high toughness and elongation at break, the UHMWPE fabric absorbed energy through extensive plastic deformation. Fracture failure was observed at the clamping boundary of the UHMWPE fabric in tests 7 and 11. The phenomenon could be attributed to the shock wave inducing a substantial bulge in the central region of the hybrid multi-ply fabric toward the back side. The central area of the UHMWPE fabric exhibited severe stretching. And the resulting tensile stress was transmitted through the warp and weft directions, generating substantial in-plane stresses at the clamping boundaries. When the UHMWPE fabric was stretched along the thickness direction (i.e., the shock wave propagation direction), the Poisson effect caused the fabric to attempt in-plane contraction. However, this contraction was constrained by the clamping boundary, inducing additional in-plane tensile stresses. A similar “pull-in” behavior was also reported in Dyneema HB26 panels under blast loading in Ref. [48], and this phenomenon became more pronounced with increasing impulse. Furthermore, shock wave reflection at the back side intensified local stresses at the clamping boundaries. In test 9, the UHMWPE fabric experienced severe perforation failure. The shock wave energy induced extreme global deformation of the hybrid multi-ply fabric. A necking effect developed in the central region of the UHMWPE fabric, causing rapid stretching, thinning, and tearing of the specimen, ultimately leading to perforation failure. A torn morphology with irregular edges was exhibited by the perforation in the UHMWPE fabric, which was fundamentally distinct from the brittle perforation observed in carbon fabrics. It confirmed that the UHMWPE fabric layer had exhausted the plastic deformation capacity.

#### 3.3.4. Effect of Fabrics on Overpressure Attenuation

Existing experimental results indicated that there is a certain relationship between the peak overpressure of the shock wave and the scaled distance. According to Ref. [49], the scaled distance could be expressed as follows:

(7) $$R=SoD/(W^{1/3})$$

where *SoD* is standoff distance (m), and *W* is the mass of charge (kg).

The peak overpressure is a widely recognized indicator for measuring the magnitude of the shock wave. As detailed in Section 2.4.2, discussing the test setup, where overpressure data were recorded using sensors positioned both in front of and behind the fabric specimens. This section will analyze the phenomenon of peak overpressure attenuation observed in the test results. To ensure consistency, the starting time of the curves measured by the overpressure sensors was synchronized to the same time, and the other parameters remained unchanged.

**Figure 26:**
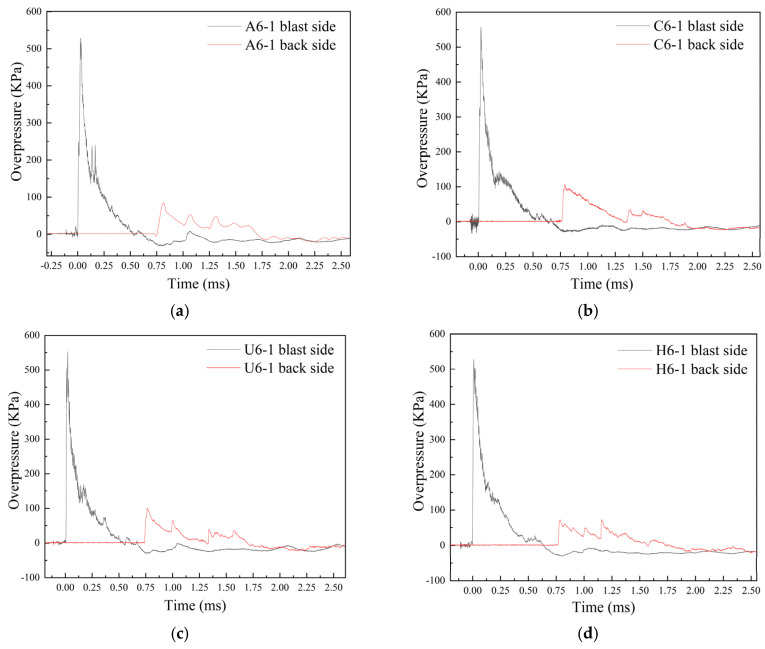
Overpressure time history curves of different tests measured by overpressure sensors; (**a**) A6-1, (**b**) C6-1, (**c**) U6-1, (**d**) H6-1.

The overpressure time history curves of the blast and back side of A6-1, C6-1, U6-1, and H6-1, respectively, are shown in Figure 26. It could be observed that the overpressure waveform of the shock wave is a triangular pulse characterized by short duration and high peak value. Additionally, a secondary peak was displayed in the curve, which was attributed to the reflection of the shock wave by the specimen. The peak overpressure, shock wave arrival time, and positive pressure duration on the blast and back side were listed in Table 5. The peak overpressure values on the blast side of the specimens range from 527.9 to 556.6 KPa. These variations may be due to the inherent differences in the TNT charges used and the unique detonation processes observed during the blast test. The overpressure attenuation percentages, which are 86.5%, 84.0%, 81.8%, and 80.8% for tests 10 (H6-1), 1 (A6-1), 3 (U6-1), and 2 (C6-1), respectively, were accompanied by corresponding time delays of 0.777 ms, 0.786 ms, 0.742 ms, and 0.723 ms. Notably, among the three single-material fabric tests under the same conditions, the maximum attenuation percentage of 84.0% and the longest time delay of 0.786 ms are observed in test 1, exhibiting the best effect on overpressure attenuation.

The different overpressure attenuation behaviors of the fabrics are due to mechanical properties and failure mechanisms. The aramid fabric exhibited perforation failure of the partial layer and yarn pull-out with damage confined to the layer on the blast side, while maintaining structural integrity in subsequent layers. Through wave reflection and inter-yarn interaction, the aramid fabric achieved better overpressure attenuation, effectively preventing shock wave propagation. In contrast, the high stiffness of the carbon fabric facilitated initial wave reflection. However, high stiffness and low toughness led to central perforation failure, permitting direct transmission of the shock wave and resulting in the worst overpressure attenuation effect. Despite high peak stress and toughness, the UHMWPE fabric showed progressive perforation failure with stress concentration induced at the perforation edges, following the penetration of initial layers. The effect was further exacerbated by free-edge shrinkage, which promoted shock wave diffraction toward the back side.

As the investigation of hybrid multi-ply fabrics with different fabric thicknesses revealed, the overpressure attenuation capacity was influenced by the fabric thickness. The overpressure attenuation percentages for H3-6, H6-1, and H9-2 are demonstrated in Table 6. The results indicated that as fabric thickness increased, the effect of fabric thickness on overpressure attenuation became more pronounced, particularly in tests 10 and 13, where no perforation failure occurred. Notably, regardless of whether perforation failure occurred in the fabric, the peak overpressure on the back side decreased by more than 80% compared to the blast side. The value of overpressure attenuation could rise to over 86% in tests 10 and 13 without perforation failure.

## 4. Conclusions

To investigate the mechanical properties and blast resistance of high-performance aramid, carbon, and UHMWPE fiber fabrics, the properties were measured through quasi-static and dynamic tensile tests. Subsequently, a near-field air blast test was conducted to research the dynamic response, failure modes, and attenuation overpressure of single-material and hybrid multi-ply fabrics. The blast resistance mechanism and overpressure attenuation effect of aramid, carbon, UHMWPE fabrics, and hybrid multi-ply fabric were revealed. The following specific conclusions could be summarized as follows:

(1) Quasi-static and dynamic tensile tests revealed different stress–strain behavior of fabrics. Carbon fabrics exhibited high stiffness but strain-rate insensitivity and brittle failure, while aramid and UHMWPE fabrics demonstrated an obvious strain-rate effect. Furthermore, UHMWPE fabrics were characterized by exceptional ductility and toughness, facilitating energy absorption through plastic deformation, whereas aramid fabrics showed more balanced stiffness and toughness.
(2) Under the blast loading, there are mainly four kinds of failure modes: perforation failure, perforation failure of partial layers, fracture, and yarn breakage or slippage. Carbon fabric suffered from brittle perforation failure due to high wave impedance and low toughness. In contrast, UHMWPE fabric absorbed energy through plastic deformation but experienced progressive perforation failure with low wave impedance and high toughness. Aramid fabric exhibited localized perforation failure of the first layer, accompanied by energy absorption mechanisms that involve yarn fibrillation and pull-out, manifesting balanced stiffness and toughness.
(3) Hybrid multi-ply fabric achieved the least failure, characterized by only perforation failure of the first layer. The configuration made use of the advantages of each fabric material. Aramid fabric layers utilized the relatively high wave impedance for initial shock wave reflection on the blast side. The carbon fabric layers depended on high stiffness for stress homogenization at the intermediate position, and the UHMWPE fabric layers exploited superior ductility for plastic energy absorption on the back side. The design effectively mitigated the weaknesses of single-material fabrics while leveraging respective advantages, creating an effective blast-resistant configuration.
(4) All single-material fabrics attenuated the shock wave with peak overpressure reductions exceeding 80%. Aramid fabric achieved no perforation failure due to the balanced stiffness and toughness, demonstrating better attenuation of overpressure ability among single-material fabric specimens. In addition, the overpressure attenuation ability of the hybrid multi-ply fabric was higher than that of single-material fabrics. And the overpressure attenuation effect of the hybrid multi-ply fabric was promoted with the increase in thickness.

## Figures and Tables

**Table 1:** Main material parameters of fabrics.

Material	Aramid Fabric	Carbon Fabric	UHMWPE Fabric
Fabric designation	F-268	HF30S-12K	ZTZ-24
Yarn linear density (g/1000 m)	166	800	126 ± 10
Yarn bulk density (g/cm^3^)	1.44	1.8	0.97
Yarn elongation at break (%)	≥3.2	1.7–2.2	3–3.5
Yarn tensile modulus (GPa)	≥125	245–270	105–110
Fabric thickness (mm)	0.3	0.5	0.5
Fabric areal density (g/m^2^)	210–220	480	235–245

**Table 2:** Blast test conditions.

Test Number	Test Name	Specimen Thickness (mm)	SoD (mm)	Charge (g)
1	A6-1	6	100	60
2	C6-1		100	60
3	U6-1		100	60
4	H3-1	3	300	10
5	H3-2		300	20
6	H3-3		200	60
7	H3-4		150	60
8	H3-5		150	20
9	H3-6		100	60
10	H6-1	6	100	60
11	H6-2		100	100
12	H9-1	9	100	100
13	H9-2		100	60

**Table 3:** Parameters related to the wave impedance of fabrics.

Material	Yarn Bulk Density (g/cm^3^)	Ed (GPa)	C (m/s)	Z (10^6^ kg/(m^2^·s))
Aramid Fabric	1.44	170	10,865	15.6
Carbon Fabric	1.8	299	12,888	23.2
UHMWPE Fabric	0.97	76	8851	8.6

**Table 4:** Perforation failure of hybrid multi-ply fabric specimens.

Test Number	Test Name	Thickness of Specimens/mm	SoD (mm)	Charge (g)	Scale Distance (m/kg^1/3^)	Perforation Failure
4	H3-1	3	300	10	1.392	A(N) C(N) U(N)
5	H3-2		300	20	1.105	A(N) C(N) U(N)
6	H3-3		200	60	0.511	A(N) C(N) U(N)
7	H3-4		150	60	0.383	A(N) C(N) U(N)
8	H3-5		150	20	0.553	A(N) C(N) U(N)
9	H3-6		100	60	0.255	A(P) C(P) U(P)
10	H6-1	6	100	60	0.255	A(P) C(N) U(N)
11	H6-2		100	100	0.215	A(P) C(P) U(P)
12	H9-1	9	100	100	0.215	A(P) C(P) U(N)
13	H9-2		100	60	0.255	A(N) C(N) U(N)

‘P’: perforation; ‘N’: non-perforation.

**Table 5:** Peak overpressure, arrival time, and positive pressure duration measured in tests.

Test Number	Test Name	Peak Overpressure (KPa)		Attenuation Percentage	The Time Reach the Peak (ms)		Delay (ms)	Positive Pressure Duration (ms)		Difference Percentage
		Blast Side	Back Side		Blast Side	Back Side		Blast Side	Back Side	
10	H6-1	527.9	71.4	86.5%	0.010	0.787	0.777	0.636	0.792	24.4%
1	A6-1	528.7	84.4	84.0%	0.028	0.814	0.786	0.521	0.894	71.6%
3	U6-1	552.8	100.7	81.8%	0.022	0.764	0.742	0.539	0.954	77.2%
2	C6-1	556.6	106.6	80.8%	0.025	0.748	0.723	0.625	0.949	51.9%

**Table 6:** Overpressure attenuation effect of a hybrid multi-ply fabric with different thicknesses.

Test Number	Test Name	SoD (mm)	Charge (g)	Peak Overpressure of Blast Side (KPa)	Peak Overpressure of Back Side (KPa)	Attenuation Percentage
9	H3-6	100	60	584.3	94.8	83.8%
10	H6-1			527.9	71.4	86.5%
13	H9-2			552.2	59.6	89.2%

## Data Availability

The data presented in this study are available on request from the corresponding author due to the anti-explosion container involved in the experimental data are related to public security.
